# Artificial Intelligence in Gastrointestinal Endoscopy and Hemostatic Decision-Making: Current Evidence, Clinical Implications and Implementation Barriers

**DOI:** 10.3390/life16050845

**Published:** 2026-05-20

**Authors:** Olga Brusnic, Adrian Boicean, Cristian Ichim, Paula Anderco, Danusia Onisor

**Affiliations:** 1 Department of Internal Medicine VII, George Emil Palade University of Medicine, Pharmacy, Science and Technology of Targu Mures, Gheorghe Marinescu Street No. 38, 540136 Targu Mures, Romania; brusnic_olga@yahoo.com (O.B.); halalisan5@yahoo.com (D.O.); 2 Faculty of Medicine, Lucian Blaga University of Sibiu, 550024 Sibiu, Romania; cristian.ichim@ulbsibiu.ro

**Keywords:** artificial intelligence, endoscopic hemostasis, gastrointestinal bleeding, machine learning

## Abstract

Artificial intelligence (AI) is increasingly transforming gastrointestinal endoscopy by supporting lesion detection, lesion characterization, quality assessment, and clinical risk prediction. Hemostatic decision-making represents a particularly complex field for AI integration, as therapeutic decisions are often made rapidly in the presence of active bleeding, impaired visualization, unstable patients, and variable lesion accessibility. This review critically examines the current evidence for AI-assisted decision-making in gastrointestinal endoscopy and endoscopic hemostasis, with emphasis on gastrointestinal bleeding, prediction of hemostatic therapy requirements, bleeding-risk stratification, rebleeding prediction, transfusion support, and post-procedural monitoring. Available studies suggest that machine learning and deep learning models may outperform conventional scoring systems in selected retrospective or validation cohorts, improve recognition of high-risk lesions, support less experienced endoscopists, and contribute to more individualized management of non-variceal bleeding, variceal bleeding, and capsule endoscopy findings. However, prospective interventional evidence remains sparse, and most available models are limited by retrospective design, single-center datasets, incomplete external validation, black-box decision-making, heterogeneous reporting, workflow barriers, and uncertain cost-effectiveness. AI should therefore be regarded as an adjunctive decision-support tool rather than an autonomous replacement for clinical judgment. Its future value will depend on prospective multicenter validation, explainability, real-time usability, regulatory clarity, post-deployment surveillance, and evidence of improved patient-centered outcomes before widespread implementation in emergency endoscopy practice.

## 1. Introduction

In recent years, medical interest in artificial intelligence (AI) has expanded considerably, particularly in specialties that depend on image interpretation and pattern recognition. The most visible progress has been reported in radiology, gastroenterology (especially endoscopy), surgery, and dermatology, where AI systems are increasingly explored as tools for detection, classification, and clinical decision support [1,2]. In gastrointestinal (GI) endoscopy, this development is particularly relevant because diagnostic and therapeutic decisions frequently depend on real-time visual interpretation, procedural quality, and rapid clinical judgment.

In GI endoscopy, AI has moved from experimental image analysis toward clinically oriented applications, including lesion detection, lesion characterization, quality assessment, and prediction of relevant outcomes [3,4]. Upper GI endoscopy is particularly suitable for its integration because diagnostic accuracy often depends on visual recognition, completeness of mucosal inspection, operator experience, and real-time interpretation of subtle endoscopic findings [5]. These features are especially important in bleeding-related scenarios, where incomplete visualization, active blood, clots, patient instability, and time pressure may affect both lesion recognition and therapeutic decision-making.

Endoscopic hemostasis represents a particularly demanding clinical context because decisions must often be made rapidly in the presence of active bleeding, impaired visualization, patient instability, and variable lesion accessibility [6,7]. In non-variceal upper GI bleeding, early risk stratification, accurate recognition of high-risk bleeding stigmata, and timely selection of endoscopic therapy are essential for reducing persistent bleeding, rebleeding, and adverse outcomes [8].

In this setting, AI may support several stages of the hemostatic pathway, including pre-endoscopic triage, prediction of the need for urgent endoscopy, recognition of high-risk lesions, estimation of hemostatic therapy requirement, transfusion support, and post-procedural monitoring [9,10,11]. Recent evidence suggests that machine learning models may improve the assessment and management of acute GI bleeding by supporting risk prediction, intervention planning, and individualized decision-making, but most applications remain at an early stage of clinical validation and implementation [12].

Although not all AI applications in GI endoscopy are hemostasis-specific, several broader endoscopic functions remain relevant to bleeding-related care. AI-assisted lesion detection, lesion characterization, and quality assessment may help reduce operator-dependent variability, improve recognition of subtle or high-risk findings, and support more consistent documentation during both routine and emergency endoscopic assessment [13,14,15]. However, these indirect applications should be distinguished from AI tools designed specifically for bleeding-risk stratification, prediction of hemostatic therapy, transfusion support, or rebleeding assessment.

Accordingly, AI should be interpreted as an adjunctive decision-support tool rather than an autonomous substitute for expert judgment. Its current value lies primarily in standardizing assessment, supporting risk prediction, and assisting clinical decision-making, while its future role will depend on prospective validation, explainability, integration into emergency workflows, and evidence of improved patient-centered outcomes [16,17].

This review critically evaluates the current evidence supporting AI-assisted decision-making in gastrointestinal endoscopy and hemostatic care, with emphasis on lesion detection, bleeding-risk stratification, prediction of hemostatic therapy requirement, rebleeding assessment, transfusion support, and barriers to clinical implementation.

## 2. Search Strategy

This structured narrative review evaluated current evidence on artificial intelligence applications in gastrointestinal endoscopy and hemostatic decision-making. The literature search was performed in PubMed/MEDLINE, Web of Science, Scopus, and Google Scholar for publications available up to April 2026. Search terms included combinations of “artificial intelligence”, “machine learning”, “deep learning”, “computer-aided detection”, “computer-aided diagnosis”, “gastrointestinal endoscopy”, “upper gastrointestinal bleeding”, “peptic ulcer bleeding”, “variceal bleeding”, “endoscopic hemostasis”, “hemostatic therapy”, “rebleeding”, “transfusion”, “clinical decision support” and “explainable artificial intelligence.”

Eligible publications included original studies, validation studies, systematic reviews, meta-analyses, guidelines, and consensus statements relevant to gastrointestinal bleeding, endoscopic hemostasis, bleeding-risk stratification, prediction of hemostatic therapy, rebleeding, transfusion support, or implementation barriers. Non-GI studies, purely technical reports without clinical relevance, non-peer-reviewed articles, conference abstracts with insufficient clinical information, and non-English publications were excluded. Additional articles were identified through reference-list screening.

After eligibility screening, 137 references were considered eligible and included in the final narrative synthesis. Eligibility was determined primarily from a clinical and hemostatic decision-making perspective. Publications were retained when they addressed gastrointestinal bleeding, endoscopic hemostasis, bleeding-risk stratification, prediction of hemostatic or endoscopic intervention, rebleeding, transfusion support, mortality prediction, or implementation barriers relevant to clinical decision support.

**Figure 1:**
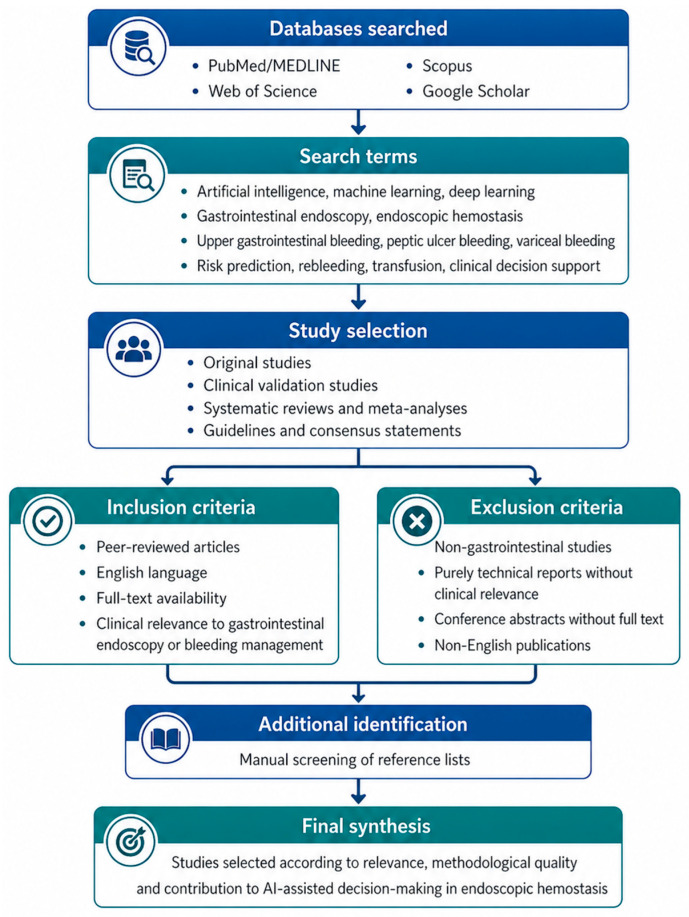
Structured literature search strategy.

Studies were prioritized according to their clinical relevance to hemostatic decision-making. Evidence directly related to gastrointestinal bleeding and endoscopic hemostasis was summarized in Table 1, while broader AI applications in gastrointestinal endoscopy were included only when relevant to lesion recognition, procedural quality, operator variability, or therapeutic decision-making. Methodological limitations were assessed descriptively, including study design, external validation, explainability, and implementation readiness. The search and selection process is summarized in Figure 1.

## 3. Conceptual Foundations of Artificial Intelligence in Endoscopic Decision Support

Artificial intelligence (AI) refers to a computer’s capacity to perform cognitive tasks typically associated with humans, particularly learning, interpretation, and decision-making [29,30]. In medical applications, AI systems may be rule-based, relying on predefined algorithms, or data-driven, relying mainly on machine learning models that identify patterns from clinical or image-based datasets [31]. Machine learning models are designed to learn from available data and generate outputs that can be applied to new, previously unseen cases [31,32,33].

In gastrointestinal endoscopy, the most relevant AI approaches include machine learning and deep learning. Conventional machine learning generally depends on predefined clinical or image-derived features, whereas deep learning can automatically extract relevant visual features from endoscopic images, most often through convolutional neural networks [34,35,36]. This is particularly relevant in endoscopic decision support, where lesion recognition, bleeding-risk estimation, and prediction of therapeutic need may depend on complex combinations of visual findings, clinical variables, and laboratory parameters.

For hemostatic decision-making, AI tools can be broadly divided into image-based models and clinical prediction models. Image-based models may support recognition of high-risk bleeding stigmata, variceal features, ulcers, vascular lesions, or potentially hemorrhagic lesions on capsule endoscopy. Clinical prediction models may integrate vital signs, comorbidities, medication exposure, laboratory parameters, risk scores, and endoscopic findings to estimate outcomes, such as need for hemostatic intervention, rebleeding, transfusion, or mortality [37,38].

However, deep learning systems also have important limitations. Many models function as “black boxes,” meaning that their internal reasoning is difficult to interpret despite known inputs and outputs [39,40]. This lack of transparency raises scientific, ethical, and legal concerns, because clinical decisions require not only accurate predictions but also understandable explanations [41,42]. Explainability methods, including heat maps, activation maps, and input-modification techniques, may improve transparency by showing which image regions or variables influenced model output [43]. Nevertheless, in emergency bleeding scenarios, AI outputs should be interpreted as decision-support signals rather than definitive therapeutic instructions.

## 4. Artificial Intelligence in Endoscopy

In endoscopy, AI applications are commonly organized into three clinically relevant domains: computer-aided detection (CADe), computer-aided diagnosis (CADx), and computer-aided quality assessment (CADq) [44,45]. CADe supports the identification of abnormal findings, CADx assists lesion characterization, and CADq evaluates procedural quality, completeness of examination, and standardization of documentation. In upper GI endoscopy, these domains follow the clinical workflow from mucosal visualization to lesion recognition, interpretation, and therapeutic decision-making [46].

Although many early AI systems were developed for colorectal polyp detection and other diagnostic indications, the same technical principles are relevant to bleeding-related care when they support rapid recognition of abnormal mucosal findings, vascular lesions, ulcers, blood clots, or small-bowel bleeding sources [29,47]. In hemostatic decision-making, the value of AI therefore extends beyond diagnosis, because lesion recognition, risk stratification, and therapeutic prioritization may directly influence the timing and type of intervention.

CADx systems may further assist endoscopists by classifying detected lesions into clinically meaningful categories, including features relevant to malignancy, invasion depth, lesion extent, or technical treatment difficulty [48,49]. Although these applications are not always hemostasis-specific, they may inform therapeutic planning when bleeding occurs in complex lesions or when the distinction between benign, malignant, and high-risk lesions influences subsequent management.

CADq tools are also indirectly relevant to emergency endoscopy because missed lesions, incomplete mucosal visualization, and inconsistent documentation may affect downstream management. AI-based quality tools have been used to support mucosal exposure, landmark recognition, blind-spot detection, bowel preparation assessment, and standardized photo-documentation [50,51,52,53,54,55,56,57]. In bleeding scenarios, these functions may help reduce operator-dependent variability, especially when visualization is impaired by blood clots, bubbles, or rapid scope movement.

Broader AI applications in upper GI endoscopy, including neoplasia detection, targeted biopsy support, H. pylori assessment, and anatomical landmark recognition, remain clinically relevant but should be distinguished from tools designed specifically for gastrointestinal bleeding or hemostatic decision-making [58,59,60,61]. In this review, these applications are considered only insofar as they illustrate how AI may improve lesion recognition, procedural quality, and consistency of endoscopic interpretation.

**Figure 2:**
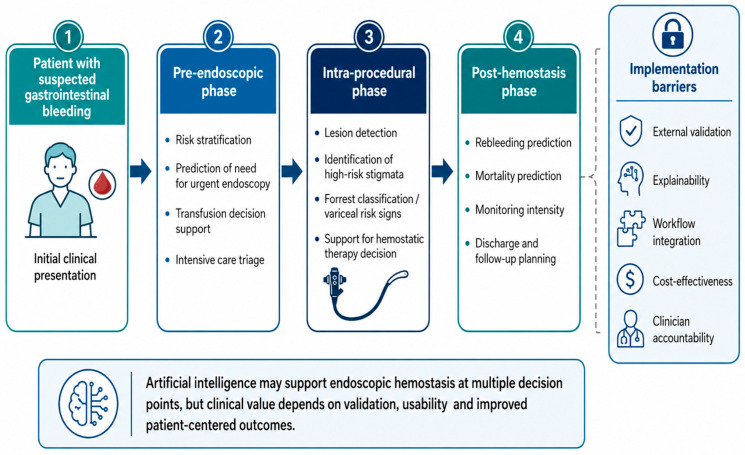
Proposed role of artificial intelligence across the endoscopic hemostasis pathway.

Taken together, these applications can be integrated into a broader clinical pathway in which AI supports decision-making before, during, and after endoscopic hemostasis, from initial risk stratification to post-procedural monitoring, as illustrated in Figure 2.

The main studies evaluating AI applications relevant to GI bleeding, hemostatic decision-making, bleeding-risk stratification, and post-procedural outcomes are summarized in Table 1.

## 5. Disease-Specific Applications of Artificial Intelligence

**Figure 3:**
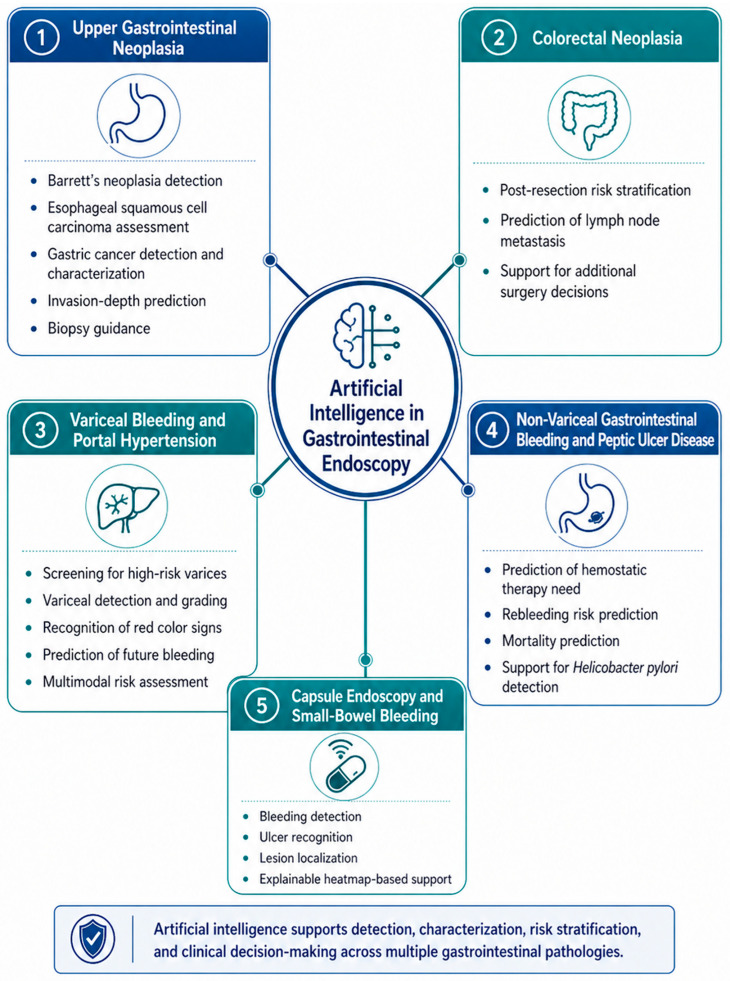
Disease-specific applications of artificial intelligence in gastrointestinal endoscopy and bleeding-related decision-making.

The main disease-specific applications of AI relevant to GI endoscopy, bleeding-related disorders, and therapeutic decision-making are summarized in Figure 3. Applications directly related to GI bleeding and hemostasis are discussed separately from broader endoscopic applications that are only indirectly relevant to hemostatic care.

### 5.1. Upper Gastrointestinal Neoplasia

These applications are included only as indirectly relevant to hemostatic decision-making, particularly when lesion characterization, invasion-depth assessment, or therapeutic planning may influence the management of bleeding, potentially hemorrhagic or technically complex upper gastrointestinal lesions [62,63].

AI-assisted CADx systems may help distinguish neoplastic Barrett’s lesions from non-dysplastic mucosa and guide targeted biopsies, but in this review, their relevance is limited to situations in which lesion characterization may influence bleeding-related therapeutic planning [64,65].

For esophageal squamous cell carcinoma, AI systems have been used to estimate invasion depth and differentiate superficial from deeply invasive disease, with accuracy comparable to expert endoscopists in some studies [66,67]. Similar models applied to gastric cancer have also shown good performance in assessing invasion depth and distinguishing malignant from benign mucosal lesions [68,69,70].

Esophageal squamous cell carcinoma remains highly lethal, particularly when diagnosed at an advanced stage, making early recognition a priority [71]. AI-based systems have demonstrated high sensitivity for detecting early lesions, often outperforming non-expert endoscopists and reaching performance levels close to experienced specialists [72].

Additional AI-supported methods, including high-resolution microendoscopy, narrow-band imaging analysis, and endocytoscopy-based models, have shown strong diagnostic potential [73,74,75,76]. However, performance may decrease when models trained on static images are applied to real-time video, mainly because of false-positive findings, indicating the need for further training and validation [77].

Gastric cancer remains a leading cause of cancer-related mortality, making early recognition of premalignant and malignant gastric lesions essential for improving clinical outcomes [78]. AI-based systems have demonstrated high sensitivity in gastric cancer detection, although false-positive results may occur in the presence of chronic atrophic gastritis or intestinal metaplasia [79]. More advanced approaches using magnifying endoscopy with narrow-band imaging have shown particular promise for early gastric cancer detection, achieving performance comparable to that of expert endoscopists [80,81]. In addition, these models can localize suspicious mucosal areas, thereby improving interpretability and increasing their potential usefulness as decision-support tools in clinical practice. AI assistance also improved diagnostic performance among both junior and senior physicians, suggesting its value as a decision-support tool rather than a replacement for endoscopists [81]. Similar models have also been used to assess the depth of gastric cancer invasion, which may help guide treatment selection between endoscopic and surgical approaches [82].

Other AI models have improved the recognition of chronic atrophic gastritis, differentiation between cancer and gastritis, and classification of early gastric mucosal lesions during magnifying endoscopy [83,84,85,86,87]. When combined with magnifying endoscopy and narrow-band imaging, AI can reach senior endoscopist-level performance and may improve the diagnostic accuracy of less experienced clinicians [81].

In gastric disease assessment, AI models have been developed to distinguish gastric neoplasms, gastric cancer, and gastric ulcers on endoscopic images [88,89]. Expanding training datasets with ulcer images markedly improved diagnostic accuracy, while broader systems have shown strong potential in detecting early gastric cancer, estimating invasion depth, and assessing differentiation status, supporting follow-up and decision-making after bleeding episodes [90].

### 5.2. Colorectal Neoplasia

Colorectal neoplasia is discussed only as an adjacent therapeutic endoscopy application rather than a direct hemostasis-specific indication. Its relevance to this review is limited to post-endoscopic therapeutic decision-making, particularly when AI may help identify patients who can safely avoid additional surgery after endoscopic treatment [91]. In T1 colorectal cancer, en bloc endoscopic resection may be sufficient when there are no clear signs of deep invasion, but lymph node metastasis is still present in about 10% of cases, creating uncertainty about the need for subsequent surgical resection with lymph node dissection [92,93,94].

To address this issue, an AI model was developed using data from 690 patients with T1 colorectal cancer, including 45 clinicopathological variables such as demographic features, comorbidities, endoscopic appearance, and histological findings [95]. Compared with American, European, and Japanese guideline-based criteria, the AI system reduced unnecessary additional surgery caused by falsely classifying lymph node-negative patients as high-risk, without missing cases with lymph node metastasis [95]. These findings suggest that AI could assist interventional endoscopists in making faster and more individualized therapeutic decisions after endoscopic resection.

### 5.3. Variceal Bleeding and Portal Hypertension

AI-based methods, including ML, neural networks, radiomics, and multimodal models, have increasingly been applied to the assessment of esophageal varices and bleeding risk in patients with liver cirrhosis. Models based on clinical, laboratory, elastography, ultrasound, computed tomography, and endoscopic data have shown better performance than several conventional scores and criteria, including Baveno VI and traditional endoscopic classifications, while helping identify patients who may require closer monitoring or treatment [96,97,98,99,100,101,102].

Newly developed tools for detecting esophageal varices have shown performance comparable to endoscopy, although larger validation studies remain necessary [81]. ENDOANGEL-GEV, for example, outperformed many endoscopists in variceal detection and improved the recognition of high-risk features [97,103]. The EVendo score has also shown potential for safely reducing unnecessary endoscopies compared with the Baveno VI criteria by estimating the likelihood of varices and varices requiring treatment using clinical and laboratory variables [104,105].

Deep learning systems using endoscopic images have also demonstrated strong potential for detecting esophageal and gastric varices, grading their severity, and recognizing red color signs. Advanced systems such as AI-assisted endoscopic platforms achieved performance comparable to endoscopists for variceal detection and were superior in identifying certain high-risk features, especially red color signs [12,106].

AI has been further explored for predicting variceal bleeding within one year. Image-based models were able to estimate bleeding risk from endoscopic findings and, when used as assistance tools, improved the diagnostic accuracy of endoscopists [22]. Multimodal models combining endoscopic images with clinical and laboratory data achieved even stronger predictive performance, suggesting that integrated AI systems may offer more individualized risk stratification [107].

Computed tomography-based AI has also shown value in assessing portal hypertension and high-risk esophageal varices. Automated spleen volume analysis, combined with platelet count, may help identify patients at increased risk and support non-invasive evaluation before or alongside endoscopic assessment [108].

### 5.4. Non-Variceal and Small-Bowel Gastrointestinal Bleeding

In non-variceal upper GI bleeding, particularly peptic ulcer bleeding, clinical decision-making depends on the accurate recognition of high-risk stigmata, estimation of rebleeding risk, and timely selection of endoscopic therapy [8]. AI-based systems may support this process by standardizing lesion assessment, identifying patients likely to require hemostatic intervention, and assisting post-procedural risk stratification [109].

ML models have been used to improve mortality prediction in patients with GI bleeding, showing better performance than conventional intensive care risk scores and helping identify low-risk patients who may not require prolonged intensive care monitoring [110]. Other AI models combining baseline clinical data with serial laboratory parameters have also predicted long-term mortality after peptic ulcer bleeding and helped identify relevant risk factors for personalized management [111,112].

AI has also been applied to recurrence prevention after peptic ulcer bleeding by supporting the detection of *Helicobacter pylori*, one of the main preventable risk factors alongside nonsteroidal anti-inflammatory drugs and low-dose aspirin [113]. In endoscopic diagnosis, deep learning systems have shown performance comparable to human endoscopists for *Helicobacter pylori* detection.

AI has shown strong performance in detecting GI bleeding and ulcers on capsule endoscopy images [114,115]. These systems can also visually indicate the suspected bleeding or ulcerated areas, making the results easier to interpret [116].

Explainable AI methods, such as heatmaps and activation maps, have also been used to identify the image regions that influenced the model’s decision [117,118,119]. This improves clinical transparency and may support lesion localization, although some systems still detect abnormalities without clearly differentiating between lesion types [118].

## 6. The Hype: Limitations, Validation Gaps, and Implementation Barriers

**Figure 4:**
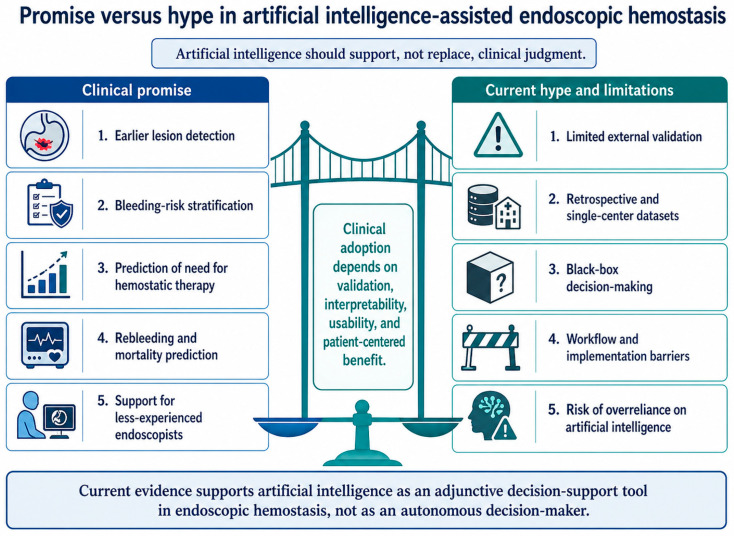
Potential benefits of artificial intelligence in endoscopic hemostasis and the main limitations, validation gaps, and implementation barriers affecting clinical adoption.

Despite the rapid expansion of AI in GI endoscopy, its translation into endoscopic hemostasis remains more limited than its technical performance might suggest [120]. High sensitivity, specificity, or area-under-the-curve values obtained in development datasets should not be interpreted as proof of clinical benefit [121]. In acute GI bleeding, the key question is not only whether an algorithm can recognize a lesion or predict risk, but whether its use changes patient management, reduces delayed or unnecessary procedures, prevents rebleeding, improves survival, or optimizes resource allocation [122]. This distinction is essential because many AI studies in medicine still evaluate diagnostic or predictive performance rather than clinically meaningful outcomes [123,124]. The balance between the potential benefits of AI and the main barriers to its clinical implementation in endoscopic hemostasis is illustrated in Figure 4.

A major limitation is the persistent gap between model development and external validation. Many ML models for GI bleeding have been derived from retrospective cohorts, administrative databases, single-center registries, or selected image datasets [125,126]. Such models may perform well in the environment in which they were trained, but their reliability may decrease when applied to different hospitals, endoscopy platforms, patient populations, bleeding etiologies, or clinical workflows. This is particularly relevant in endoscopic hemostasis, where real-world decisions are influenced by hemodynamic instability, anticoagulant exposure, comorbidities, lesion accessibility, operator experience, and device availability—variables that are not always fully captured in model development [127,128].

Generalizability is further threatened by dataset shift and data drift. Endoscopic images obtained in emergency bleeding conditions differ substantially from curated training datasets: blood, clots, bubbles, poor insufflation, unstable visualization, active peristalsis, suboptimal lighting, and rapid scope movement can all modify image quality and model input [129]. Similarly, clinical prediction models may degrade when laboratory practices, transfusion policies, hemostatic devices, admission thresholds, or patient case-mix change over time [130]. Without prospective monitoring after deployment, an AI system that was accurate at validation may become unreliable during routine clinical use.

Another source of hype is incomplete and heterogeneous reporting. AI studies may omit essential details such as algorithm version, preprocessing methods, exclusion criteria for poor-quality inputs, threshold selection, code accessibility, calibration, handling of missing data, or subgroup performance. This weakens reproducibility and makes it difficult to compare models across studies. The emergence of reporting frameworks such as CONSORT-AI, TRIPOD + AI, DECIDE-AI, and STARD-AI reflects the need for higher methodological standards before AI tools are considered clinically mature [124,126,131,132].

The “black box” nature of many deep learning systems remains a major barrier in hemostatic decision-making [133]. In GI bleeding, an incorrect recommendation may delay urgent therapy, lead to unnecessary intervention, or falsely reassure clinicians after apparently successful hemostasis [7,132]. Explainability methods such as heatmaps, activation maps, local interpretable model-agnostic explanations, and SHapley additive explanations may improve transparency, but they do not necessarily prove that the model is using clinically valid reasoning [40,131]. Visual attention on a suspicious region does not equal causal interpretation, and feature attribution does not eliminate the need for clinical judgment.

Medicolegal responsibility is another unresolved issue in AI-assisted hemostatic decision-making. In emergency bleeding scenarios, delayed therapy, unnecessary intervention, or inappropriate reassurance after apparent hemostasis may have immediate consequences for patient safety. Therefore, the final responsibility for clinical decisions should remain with the treating physician, while institutions and manufacturers must define clear rules for validation, monitoring, documentation, software updates, and accountability when AI output contributes to patient management [17,134]. AI recommendations should be documented as supportive information rather than autonomous therapeutic decisions.

Implementation is also constrained by human factors. In emergency endoscopy, AI must operate in a high-pressure environment where the endoscopist is simultaneously responsible for diagnosis, suction, irrigation, device selection, hemostatic technique, patient instability, and communication with the anesthesia or intensive care team [17,132,135]. Poorly integrated alerts, excessive false positives, delayed outputs, or unclear recommendations may increase cognitive load rather than reduce it. For this reason, early-stage evaluation of AI systems should include usability, workflow integration, safety, and clinician interaction, not only model accuracy.

A further implementation risk is overreliance on AI. The assumption that it will automatically improve clinician performance ignores the possibility of automation bias, deskilling, or reduced vigilance. Recent evidence from colonoscopy suggests that continuous exposure to AI assistance may reduce unaided endoscopist performance, raising concerns that similar effects could occur in other visually dependent endoscopic tasks [136,137]. In hemostasis, where rapid independent judgment is essential when AI fails, is unavailable, or produces uncertain outputs, preserving endoscopist expertise remains critical.

Economic and regulatory barriers are equally important, because AI systems require acquisition costs, hardware integration, maintenance, software updates, cybersecurity safeguards, data governance, training, and post-market surveillance [134]. Their value, therefore, depends not only on diagnostic performance but also on whether they reduce unnecessary endoscopies, shorten time to hemostasis, decrease rebleeding, rationalize transfusion, reduce intensive care admissions, or improve survival [17]. Without cost-effectiveness analyses and clear reimbursement pathways, even technically successful systems may remain difficult to implement on a large scale.

Therefore, current evidence supports AI as an adjunctive tool rather than an autonomous decision-maker in endoscopic hemostasis. Its clinical value will depend on prospective validation, real-time usability, transparent reporting, integration into emergency workflows, and demonstrable improvement in patient-centered outcomes.

### Limitations

Several limitations should be considered when interpreting the findings of this review. Although the literature search followed a structured approach, the evidence was synthesized narratively, with a descriptive appraisal of methodological quality rather than a formal risk-of-bias assessment. This approach was appropriate for the broad and heterogeneous nature of the topic, but it limits the ability to compare studies systematically or to quantify the overall strength of evidence. The available literature varies considerably in terms of clinical setting, patient population, AI architecture, input data, validation strategy, outcome definition, and reporting standards. As a result, models developed for lesion detection, bleeding-risk stratification, transfusion prediction, rebleeding assessment, or mortality estimation cannot be interpreted as equivalent in clinical maturity or implementation readiness.

A further limitation is that much of the current evidence remains derived from retrospective cohorts, selected image datasets, or internally validated prediction models. Prospective multicenter validation and real-time interventional studies remain limited, particularly in emergency endoscopy and hemostatic decision-making. Moreover, many studies primarily report diagnostic or predictive performance metrics, such as accuracy, sensitivity, specificity, or area under the curve, whereas fewer studies demonstrate that AI-assisted workflows improve clinically meaningful outcomes, including time to hemostasis, rebleeding prevention, transfusion optimization, resource allocation, intensive care use, or survival. Therefore, the conclusions of this review should be interpreted as a critical synthesis of an evolving evidence base rather than as definitive confirmation of clinical effectiveness. Further prospective validation, standardized reporting, and outcome-oriented trials are needed before AI-assisted hemostatic decision-making can be widely adopted in routine practice.

## 7. Conclusions

Artificial intelligence shows clear potential to support endoscopic hemostasis by improving lesion recognition, risk stratification, prediction of hemostatic therapy needs, and post-procedural monitoring. Current evidence suggests that these tools may reduce operator-dependent variability and assist clinical decision-making, particularly in complex or high-risk bleeding scenarios.

However, AI is not yet ready to replace expert judgment. Most available models still require stronger external validation, prospective testing, workflow integration, and proof of benefit on clinically relevant outcomes such as rebleeding, intervention timing, resource use, and survival. Its use in endoscopic hemostasis represents a promising adjunct rather than an autonomous solution. Its future value will depend on whether technical performance can be translated into safer, faster, and more individualized patient care.

## Figures and Tables

**Table 1:** AI-based decision support in gastrointestinal bleeding and hemostatic decision-making.

Clinical Setting	Study Design/Validation	AI Approach	Main Finding	Relevance to Hemostatic Decision-Making	Ref.
Bleeding peptic ulcer disease	Retrospective image-based study; internal testing; external validation not reported.	Deep learning classification of endoscopic images	The model classified bleeding peptic ulcer images and performed better than inexperienced endoscopists in determining whether endoscopic therapy was required.	Directly relevant, as it supports the use of AI to assist less experienced endoscopists in recognizing high-risk ulcer stigmata and therapeutic need.	[18]
Peptic ulcer bleeding	Retrospective single-center image-analysis study; external validation not reported.	Image-based analysis of Forrest classification	The study showed that high-risk and low-risk Forrest lesions have distinct visual patterns, with good agreement among endoscopists in a single-center setting.	Provides the clinical and visual foundation for AI models aimed at standardizing Forrest-based hemostatic decision-making.	[19]
Stable non-variceal upper GI bleeding	Prospective observational registry; model development and internal validation.	Prediction of mortality, hypotension, and early rebleeding	Machine learning models outperformed conventional risk scores in predicting adverse events within 7 days.	Supports the role of AI in early risk stratification before and after endoscopy.	[20]
Acute upper GI bleeding	Retrospective cohort study; internal validation.	Prediction of need for hemostatic therapy	The random forest model predicted the need for endoscopic, radiological, or surgical hemostasis more accurately than the Glasgow-Blatchford score.	Highly relevant for triage, as it identifies patients most likely to benefit from urgent hemostatic intervention.	[9]
Acute upper GI bleeding	Prospectively collected database retrospectively reviewed; internal validation.	Prediction of endoscopic intervention	The model identified patients requiring endoscopic intervention with good performance and was proposed as a tool for prioritizing referral in resource-limited settings.	Relevant for pre-endoscopic decision support and resource allocation in acute bleeding pathways.	[10]
Peptic ulcer bleeding after initial endoscopic hemostasis	Retrospective single-center cohort; internal validation.	Interpretable prediction of short-term rebleeding	An explainable model predicted rebleeding after endoscopic hemostasis and identified clinically relevant predictors, including coagulation parameters, renal markers, risk scores, and lesion location.	Directly relevant to post-hemostasis management, helping identify patients who may need closer monitoring or repeat intervention.	[21]
Esophageal varices	Multicenter retrospective cohort study; externally validated using an independent test dataset.	Prediction of 12-month variceal bleeding using endoscopic images	Deep learning predicted future variceal bleeding from endoscopic images, and AI assistance improved endoscopist accuracy.	Extends artificial intelligence-assisted bleeding prediction to variceal disease and supports image-based risk stratification.	[22]
Non-variceal upper GI bleeding in intensive care	Retrospective ICU-based cohort; internal validation.	Interpretable mortality prediction	An interpretable model predicted mortality in critically ill patients and highlighted shock, neurological status, renal disease, age, albumin, and alkaline phosphatase as major prognostic factors.	Useful for prognosis-oriented decision support in severe bleeding cases requiring intensive care.	[23]
Acute GI bleeding in intensive care	Retrospective ICU-based cohort; internal validation.	Mortality prediction	Machine learning models predicted in-hospital mortality more accurately than the Acute Physiology and Chronic Health Evaluation II score, especially when both approaches were combined.	Supports integration of AI into severity assessment for high-risk bleeding patients.	[24]
Overt GI bleeding	Prospective multicenter registry-based study; five-fold cross-validation/internal validation.	Mortality risk prediction	Machine learning models outperformed Glasgow-Blatchford and pre-endoscopic Rockall scores for in-hospital mortality prediction in a multicenter registry.	Supports the argument that AI may improve global risk assessment beyond traditional scoring tools.	[25]
Acute upper GI bleeding	Retrospective cohort; external validation cohort.	Transfusion decision support	A multitask model predicted transfusion need, blood product type, and transfusion volume, with better performance than conventional scoring systems.	A relevant adjunctive tool, such as a transfusion strategy, is closely linked to bleeding severity and hemostatic management.	[26]
Suspected mid-lower GI bleeding	Retrospective analysis of a prospective cohort; validation status reported for the AI-assisted reading system.	Artificial intelligence-assisted capsule endoscopy reading	Artificial intelligence-assisted capsule endoscopy detected more potentially hemorrhagic lesions than conventional reading and colonoscopy, with higher sensitivity and preserved specificity.	Relevant to lesion detection and reduction in missed bleeding sources, although less directly related to therapeutic hemostasis.	[27]
Superficial esophageal squamous cell carcinoma	Video-based diagnostic performance study; indirect relevance to hemostatic decision-making.	Artificial intelligence-assisted video diagnosis	AI assistance improved endoscopist diagnostic performance across different experience levels.	Indirectly relevant, as it supports the broader concept that AI can reduce operator-dependent variability in endoscopic interpretation.	[28]

## Data Availability

Not applicable.
